# A Model of Regularization Parameter Determination in Low-Dose X-Ray CT Reconstruction Based on Dictionary Learning

**DOI:** 10.1155/2015/831790

**Published:** 2015-10-04

**Authors:** Cheng Zhang, Tao Zhang, Jian Zheng, Ming Li, Yanfei Lu, Jiali You, Yihui Guan

**Affiliations:** ^1^Medical Imaging Laboratory, Suzhou Institute of Biomedical Engineering and Technology, Chinese Academy of Sciences, Suzhou 215163, China; ^2^Changchun Institute of Optics, Fine Mechanics and Physics, Chinese Academy of Sciences, Changchun 130033, China; ^3^University of Chinese Academy of Sciences, Beijing 100049, China; ^4^PET Center, Huashan Hospital, Fudan University, Shanghai 200235, China

## Abstract

In recent years, X-ray computed tomography (CT) is becoming widely used to reveal patient's anatomical information. However, the side effect of radiation, relating to genetic or cancerous diseases, has caused great public concern. The problem is how to minimize radiation dose significantly while maintaining image quality. As a practical application of compressed sensing theory, one category of methods takes total variation (TV) minimization as the sparse constraint, which makes it possible and effective to get a reconstruction image of high quality in the undersampling situation. On the other hand, a preliminary attempt of low-dose CT reconstruction based on dictionary learning seems to be another effective choice. But some critical parameters, such as the regularization parameter, cannot be determined by detecting datasets. In this paper, we propose a reweighted objective function that contributes to a numerical calculation model of the regularization parameter. A number of experiments demonstrate that this strategy performs well with better reconstruction images and saving of a large amount of time.

## 1. Introduction

Nowadays, X-ray computed tomography (CT) is still an important part of biomedical imaging technologies for the reason that the reconstructed image is of high spatial resolution and quality. Nevertheless, it confirms that an overdose of radiation possibly increases the risk of genetic or cancerous diseases, making it urgent to develop creative and effective reconstruction techniques to fit low-dose CT scanning protocol. Obviously, the X-ray flux cannot be reduced much since the signal-to-noise ratio (SNR) of measured data declines with the reduction of dose. Another approach is to decrease the number of projection angles, which will lead to incomplete few-view data. In this case, analytic-based algorithms like FDK [1], which are derived from a continuous imaging model and in need of dense sampled projections, are sensitive to insufficient projection data and arrive at a terrible result. However, algebraic algorithms like the simultaneous algebraic reconstruction technique (SART) [2] solved the problem better by transforming it to a series of linear equations.

Recently, Candes et al. [3, 4] have made compressed sensing theory popular in information theory field. This theory indicates that a variety of signals can be represented sparsely in a certain transform domain. Therefore, original signal can be recovered accurately by far fewer samples while there is no need to follow the Shannon/Nyquist sampling theorem. A principle called restricted isometry property (RIP) guarantees the perfect recovery of any sparse signal [5]. This novel theory has been applied to many regions, like information technology [6], signal and image processing [7], inverse filtering [8], and so on. It is said that the data acquisition process with compression is good for enhancing image quality because this method can increase imaging speed and suppress the artifacts caused by patients' movement [9]. For these benefits, many compressed sensing based algorithms are created to deal with few-view CT reconstruction problem. One major group is based on the total variation (TV), which takes the TV of the image as the sparse constraint. The image is determined by minimizing the TV term with the constraints of the linear projection equations. Sidky and Pan presented an improved TV-based algorithm named adaptive steepest descent projection onto convex sets (ASD-POCS) in circular cone-beam framework [10]. Another similar method called gradient projection Barzilari Borwein (GPBB) has a faster convergence speed [11]. Besides the TV minimization algorithms, dictionary learning is also helpful to sparse representation. During the reconstruction process, the image is divided into many overlapped patches, represented sparsely by overcomplete elements of a particular dictionary. Xu et al. combined statistical iterative reconstruction (SIR) with dictionary learning and got a better reconstruction result than TV-based methods in the low-dose CT condition [12]. According to Xu's paper, this method is robust to noise and obtains a better reconstructed image with more details than the TV-based methods do. Naturally, there are some parameters relevant to the final result. Some of them, like the sparse level, the scale of the dictionary, and so on, have less change due to different scanning data and then can be empirically selected. However, there is a special parameter changing according to the phantom, the scanning protocol, the noise level, and other factors. This parameter plays an important role in the reconstruction program to balance the data fidelity term and the regularization term while determining its value is time consuming with many attempts. Hence, there is no doubt that providing a model to select a proper value of this parameter according to the scanning data is essential for the algorithm based on dictionary learning, which leads to better result and time saving.

This paper is organized as follows. In Section 2, the problem of low-dose CT reconstruction is stated and the algorithm based on dictionary learning is reviewed. In Section 3, the model of regularization parameter determination is proposed by function fitting method. In Section 4, a series of experiments are performed and corresponding discussions are given. Finally, there is the conclusion at the end of this paper.

## 2. Notation and Problem Description

### 2.1. Background and Notation Interpretation

According to previous work by Xu et al. [12], SIR is united with dictionary learning to derive the algorithm. SIR assumes that the measured data can be regarded as the Poisson distribution

(1) $$\begin{array}{c} y_{i}\sim \text{Poisson}\left\{\;b_{i}e^{-l_{i}}+r_{i}\;\right\},\;i=1,\ldots ,I, \end{array}$$

where **b** = (*b*_1_, *b*_2_,…,*b*_*I*_)^*T*^ ∈ **R** ^*I*×1^ is the entrance X-ray intensity, **y** = (*y*_1_, *y*_2_,…,*y*_*I*_)^*T*^ ∈ **R** ^*I*×1^ is the exit X-ray intensity, **l** = (*l*_1_, *l*_2_,…,*l*_*I*_)^*T*^ ∈ **R** ^*I*×1^ is the integral of the linear attenuation coefficient with *l*_*i*_ = [**A** ***μ***]_*i*_ = ∑_*j*=1_^*N*^2^^ *a*_*ij*_ *μ*_*j*_, **A** = {*a*_*ij*_} ∈ **R** ^*I*×*N*^2^^ is the system matrix, the reconstructed image ***μ*** = (*μ*_1_, *μ*_2_,…,*μ*_*N*^2^_)^*T*^ is a linear attenuation coefficient distribution, which transforms the initial image of *N* × *N* pixels to a vector ***μ*** ∈ **R** ^*N*^2^×1^, and *r*_*i*_ represents the read-out noise.

The objective function of SIR is as

(2) $$\begin{array}{c} \overset{I}{\underset{i=1}{\sum }}\frac{\omega_{i}}{2}{\left(\;{\left[\;A\mu \;\right]}_{i}-{\hat{l}}_{i}\;\right)}^{2}+\lambda R\left(\;\mu \;\right), \end{array}$$

where $\varphi \left(\mu \right)=\sum_{i=1}^{I}\left(\omega_{i}/2\right){\left({\left[A\mu \right]}_{i}-{\hat{l}}_{i}\right)}^{2}$ is the data fidelity term, $\hat{l}={\left({\hat{l}}_{1},{\hat{l}}_{2},\ldots ,{\hat{l}}_{I}\right)}^{T}\in R^{I\times 1}$ is the measured data of **l** calculated by ${\hat{l}}_{i}=\ln\left(b_{i}/\left(y_{i}-r_{i}\right)\right)$, *ω*_*i*_ = (*y*_*i*_ − *r*_*i*_)^2^/*y*_*i*_ is the statistical weight, and *R*(***μ***) is the regularization term.

The regularization term usually contains prior information of the image, like sparse constraint. When the sparse representation is acquired by dictionary learning theory, we can replace *R*(***μ***) = ∑_*s*_‖**E**_*s*_ ***μ*** − **D** **α**_*s*_‖_2_^2^ + ∑_*s*_ *ν*_*s*_‖**α**_*s*_‖_0_ in the objective function. Therefore, the reconstruction problem is equivalent to the following minimization:

(3) minμ,α,D⁡∑i=1Iωi2Aμi−l^i2+λ∑sEsμ−Dαs22+∑sνsαs0,

where **E**_*s*_ = {*e*_*nj*_^*s*^} ∈ **R** ^*N*_*o*_^2^×*N*^2^^ is an operator to extract patches with *N*_0_ × *N*_0_ pixels from the image, **D** = (**d**_1_, **d**_2_,…, **d**_*K*_) ∈ **R** ^*N*_0_^2^×*K*^ is the training dictionary whose column **d**_*k*_ ∈ **R** ^*N*_0_^2^×1^ is called an atom of the same size of a patch, **α**_*s*_ ∈ **R** ^*K*×1^ has few nonzero entries as a sparse representation of patches by the dictionary basis **D**, and the variables *λ* and *ν*_*s*_ are regularization parameters. In this optimization problem, ***μ***, **α**, and **D** are all unknown; hence, a practical plan of minimizing the object function is an alternating minimization scheme. The plan divides the primary problem into two recursive steps: update of the dictionary model and update of the image. The final result is acquired by operating the two steps alternately until reaching a stopping criterion.

### 2.2. Update of the Dictionary Model

During this procedure, the image ***μ*** is supposed to be fixed, meaning that the data fidelity term is a constant. The optimization problem is simplified to the one as

(4) $$\begin{array}{c} \underset{\left(D\right),\alpha }{\min}\underset{s}{\sum }{\left\|\;E_{s}\mu^{t}-D\alpha_{s}\;\right\|}_{2}^{2}+\underset{x}{\sum }\nu_{s}{\left\|\;\alpha_{s}\;\right\|}_{0}, \end{array}$$

where ***μ*** ^*t*^ is an intermediate image of the last updating step. In the adaptive dictionary based statistical iterative reconstruction (ADSIR), the dictionary is defined dynamically based on the unknown image while the dictionary in the global dictionary based statistical iterative reconstruction (GDSIR) is predefined beforehand [12]. Previous researches have proved that the K-SVD algorithm performs well at training the dictionary [13]. Once the dictionary is determined, the OMP algorithm is used to update the sparse coding [14] with a predetermined sparse level, instead of solving the *l*_0_-norm problem as (4) directly.

### 2.3. Update of the Image

While updating the image, the dictionary and sparse coding remain invariable. In other words, the problem transforms to the form as

(5) $$\begin{array}{c} \underset{\mu }{\min}\overset{I}{\underset{i=1}{\sum }}\frac{\omega_{i}}{2}{\left(\;{\left[A\mu \right]}_{i}-{\hat{l}}_{i}\;\right)}^{2}+\lambda \underset{s}{\sum }{\left\|\;E_{s}\mu -D\alpha_{s}\;\right\|}_{2}^{2}, \end{array}$$

where *λ* is the regularization parameter balancing the data fidelity term $\sum_{i=1}^{I}\left(\omega_{i}/2\right){\left({\left[A\mu \right]}_{i}-{\hat{l}}_{i}\right)}^{2}$ and the regularization term ∑_*s*_‖**E**_*s*_ ***μ*** − **D** **α**_*s*_‖_2_^2^. The regularization term is already a separable quadratic function. By replacing the data fidelity term with a separable paraboloid surrogate [15], the optimization can be iteratively solved by

(6) $$\begin{array}{c} \mu_{j}^{t+1}={\left[\;\mu_{j}^{t}-\frac{\sum_{i=1}^{I}\left(a_{ij}\omega_{i}\left({\left[A\mu^{t}\right]}_{i}-{\hat{l}}_{i}\right)\right)+2\lambda \sum_{s}^{}\sum_{n=1}^{N_{0}^{2}}e_{nj}^{s}\left({\left[E_{s}\mu^{t}\right]}_{n}-{\left[D\alpha_{s}\right]}_{n}\right)}{\sum_{i=1}^{I}\left(a_{ij}\omega_{i}\sum_{k=1}^{N^{2}}a_{ik}\right)+2\lambda \sum_{s}^{}\sum_{n=1}^{N_{0}^{2}}e_{nj}^{s}\sum_{k=1}^{N^{2}}e_{nk}^{s}}\;\right]}_{+}\;j=1,2,\ldots ,N^{2}. \end{array}$$

## 3. Materials and Methods

### 3.1. Effect of the Regularization Parameter

As mentioned above, the regularization parameter *λ* is of great importance during the update of image (5). We consider the optimizing problem of the form

(7) μλ=arg⁡minμ ⁡φμ+λRμ,φμ=∑i=1Iωi2Aμi−li2,Rμ=∑sEsμ−Dαs22.

If there is ***μ***(*λ*_1_) = min_***μ***_⁡*φ*(***μ***) + *λ*_1_ *R*(***μ***), ***μ***(*λ*_2_) = min_***μ***_⁡*φ*(***μ***) + *λ*_2_ *R*(***μ***), 0 < *λ*_1_ < *λ*_2_, then we can get *φ*(***μ***(*λ*_1_)) ≤ *φ*(***μ***(*λ*_2_)) and *R*(***μ***(*λ*_1_)) ≥ *R*(***μ***(*λ*_2_)) by an easy derivation of the unequal relations:

(8) φμλ1+λ1Rμλ1≤φμλ2+λ1Rμλ2,φμλ2+λ2Rμλ2≤φμλ1+λ2Rμλ1.

It shows that a smaller *λ* makes the data fidelity term smaller and the regularization term bigger, which means that the sparse constraint has less effect on the optimizing process and more noise will appear in the final image. On the other hand, a bigger *λ* weakens the effect of the data fidelity term, generating a loss of some fine details in the image. For example, *λ* should be increased to suppress the noise increment in the projection domain since the data fidelity term is proportional to the noise standard deviation. In order to get an optimal result, previous work selects a great many values of *λ* and picks out the best one by comparing the final images. This testing strategy is of great time consuming, making the algorithm based on dictionary learning not friendly to the reconstruction task.

### 3.2. Morozov's Principle and the Balancing Principle

The research on the choices of regularization parameters in linear inverse problems appears early in 1998 [16]. The original optimizing function of the inverse problem is like

(9) $$\begin{array}{c} J\left(\;f,\beta \;\right)=\frac{1}{2}{\left\|\;Tf-z\;\right\|}_{Y}^{2}+\beta {\left\|\;f\;\right\|}_{X}^{2}. \end{array}$$

Take *Y* = 2, *X* = 2 as an example. If the noise level of *z* is known, one efficient tool for selecting the proper regularization parameter *β* is the well-known Morozov discrepancy principle [16]. To adaptively determine the regularization parameter, a model function is brought in [17]:

(10) $$\begin{array}{c} m\left(\;\beta \;\right)=b+\frac{s}{t+\beta }. \end{array}$$

To find a solution of *β*, (9) is rewritten as

(11) Fβ=12Tfβ−z22+βfβ22=φβ+βF′βwith  φβ=12Tfβ−z22,  F′β=fβ22.

When *β* → *∞*, it obviously shows that the solution of the minimization problem is *f*_*β*_ = 0, and then *b* = (1/2)‖*z*‖_2_^2^ is obtained easily. The other two variables *s* and *t* can be determined by the equations *m*(*β* ^*k*^) = *F*(*β* ^*k*^), *m*′(*β* ^*k*^) = *F*′(*β* ^*k*^). To solve the parameter *β* iteratively, the balancing principle [18] is introduced as

(12) $$\begin{array}{c} \left(\;\sigma -1\;\right)\varphi \left(\;\beta^{*}\;\right)=\beta^{*}F^{\prime }\left(\;\beta^{*}\;\right), \end{array}$$

where *σ* > 1 controls the relative weight of the two terms. Equation (12) can be written as

(13) $$\begin{array}{c} F\left(\;\beta^{*}\;\right)=\sigma \left(\;F\left(\;\beta^{*}\;\right)-\beta^{*}F^{\prime }\left(\;\beta^{*}\;\right)\;\right), \end{array}$$

which is a fixed point iteration. *β* ^*k*+1^ is calculated by the formula

(14) Fβk+1σFβk−βkF′βk=σmβk−βkm′βk.

Although the balancing principle behaves well in the inverse problem model, there is no direct way introducing the method to the dictionary based algorithm. The regularization term has a minimum value greater than zero, which leads to the derivation as follows:

(15) for Rμ=∑sEsμ−Dαs22≥δ>0assume Fλ=∑i=1Iωi2Aμλi−li2+λ∑sEsμλ−Dαs22=φλ+λF′λ Fλ=mλ=b+st+λwhen λ⟶∞,  b≥λδ⟶∞.

Therefore, the strategy that determining the regularization parameter adaptively accords to the last iterative result is not reasonable. We should look for a selecting strategy which can determine the proper value of the regularization parameter by making an analysis of the projection data.

### 3.3. Weight Modification of the Objective Function

In order to find out an applicable selecting model of the regularization parameter, we reconsider the minimizing problem (5) and the updating formula (6). According to the former work, the data fidelity term is replaced with a separable surrogate [15]

(16) Qμ;μt=∑i=1I ∑j=1N2αijωi2×aijαijμj−μjt+Aμti−l^i2,

where

(17) $$\begin{array}{c} \overset{N^{2}}{\underset{j=1}{\sum }}\alpha_{ij}=1\;\forall i,\;\;\alpha_{ij}\geq 0. \end{array}$$

And one convenient choice is

(18) $$\begin{array}{c} \alpha_{ij}=\frac{a_{ij}}{\sum_{j=1}^{N^{2}}a_{ij}}. \end{array}$$

By making use of the surrogate, (5) becomes a separable form

(19) $$\begin{array}{c} \mu =\arg\underset{\mu }{\min}\;Q\left(\;\mu ;\mu^{t}\;\right)+\lambda R\left(\;\mu \;\right), \end{array}$$

where *Q*(***μ***; ***μ*** ^*t*^) and *R*(***μ***) both can be represented as the sum of a series of quadratic functions, whose variables are *μ*_*j*_  (*j* = 1,2,…, *N* ^2^). After some variable exchanges, (19) can be rewritten as

(20) Qμ;μt=12∑j=1N2pjμj−cjt2+C1,Rμ=∑j=1N2qjμj−djt2+C2with  pj=∑i=1Iaijωi∑k=1N2aik,  qj=∑s∑n=1N02enjs∑k=1N2enks,  cjt=μjt−∑i=1IaijωiAμti−l^ipj,  djt=μjt−∑s∑n=1N02enjsEsμtn−Dαsnqj,  μjt+1=pjcjt+2λqjdjtpj+2λqj+.

From the above, the image updating formula is just the same as (6); the quadratic term coefficient *p*_*j*_ only depends on the system matrix **A** and the statistical weight  **ω**. We make a weight modification on the regularization term

(21) R~μ=∑j=1nrjqjμj−djt2+C2=12∑j=1npjμj−djt2+C2s.t.rj=12pjqj.

By eliminating the constant term, the image reconstruction process is equivalent to solving the following optimization problem:

(22) μarg⁡minμ⁡ Qμ;μt+λR~μ=arg⁡minμ⁡12∑j=1N2pjμj−cjt2+12λ∑j=1npjμj−djt2,

which can be solved iteratively by

(23) $$\begin{array}{c} \mu_{j}^{t+1}={\left[\;\frac{c_{j}^{t}+\lambda d_{j}^{t}}{1+\lambda }\;\right]}_{+}. \end{array}$$

As shown in (23), *λ* determines the relative impact on the updating image ***μ*** by the data fidelity term and the regularization term, respectively.

### 3.4. Evaluation Model of Regularization Parameter

Before developing the evaluation model, some discussions about the reconstruction result are displayed firstly. Once a value of *λ* is selected randomly, by solving (22) iteratively, it infers that the relative error of the data fidelity term is as

(24) $$\begin{array}{c} \delta =\frac{\sum_{i=1}^{I}\left(\omega_{i}/2\right){\left({\left[A\mu \right]}_{i}-l_{i}\right)}^{2}}{\sum_{i=1}^{I}\left(\omega_{i}/2\right)l_{i}^{2}}. \end{array}$$

The relative error depends on the phantom image, the noise level, the regularization parameter, and so on. When it comes to the situation that the regularization parameter is infinite as *λ* → *∞*, (23) and (24) will be like the following form:

(25) μjt+1=djt+,

(26) δλ→∞=∑i=1Iωi/2Aμλ→∞∗i−li2∑i=1Iωi/2li2s.t.  λ⟶∞.

It is naturally derived that the relative error *δ*_*λ*→*∞*_ increases with the increment of the noise level in projection domain. In addition, it has been mentioned above (in Section 3.1) that *λ* should be increased with the noise increment. Therefore, the proper *λ* has a monotonous relation with the parameter *δ*_*λ*→*∞*_. Since *δ*_*λ*→*∞*_ can be easily determined by operating the reconstruction algorithm based on dictionary learning once with *λ* → *∞*, the proper value of *λ* can be calculated if a reasonable function as *λ* ^*∗*^ = *f*(*δ*_*λ*→*∞*_) can be found.

With the help of a series of tests, the relationship between *λ* ^*∗*^ and *δ*_*λ*→*∞*_ is fitted by a piecewise quadratic function as follows:

(27) λ∗=1.74485δG2+0.58883δG−6.88253if  δG>1.96λ∗=−0.21545δG2+1.08602δG−0.32634if  δG≤1.96with  δG=106δλ→∞.

Finally, taking ADSIR as an example, the workflow of the developed algorithm is exhibited in Algorithm 1. In addition, the ordered subsets convex (OSC) algorithm [19] is utilized as an acceleration of the convergence.

## 4. Experimental Results and Discussion

**Figure 1:**
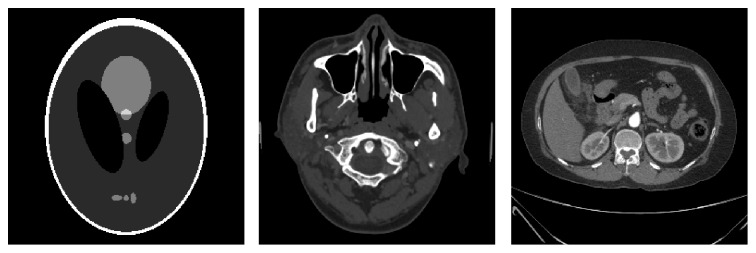
From left to right are Shepp-Logan phantom, human head slice image, and human abdomen slice image. The whole windows are [0,1], [−1000,1436] HU, and [−1000,837] HU while the display windows are [0.15,0.45], [400,1000] HU, and [−160,400] HU, respectively.

To make the evaluation of the regularization parameter possible, the developed algorithm improves ADSIR with a modified weight of the regularization term while the weight is adaptive to the data fidelity term. So the proposed algorithm is named adaptive weight regularized ADSIR (AWR-ADSIR). In this section, a series of reconstruction experiments are exhibited to validate that the regularization selecting principle in AWR-ADSIR is practical. The simulation numerical phantoms are Shepp-Logan phantom, human head slice image, and human abdomen slice image. The Shepp-Logan phantom is a numeric phantom with pixels intensities ranging from 0 to 1. The sample images of human head slice and human abdomen slice are the FBP reconstruction results based on full-sampling scanning data, which are obtained from our collaborator. All of these phantom images are of 256 × 256 pixels presented in Figure 1. The scanning data are simulated as an undersampling situation with different noise levels. Firstly, different regularization parameters are selected to demonstrate that the one chosen by the algorithm leads to the best reconstruction result. Secondly, by comparing the quality of images reconstructed by diverse algorithms, which are SART, GPBB, ADSIR, and AWR-ADSIR, it can be confirmed that AWR-ADSIR is of remarkable performance among these algorithms. Finally, the selecting principle is used in the GDSIR model, proving that it also works. All the algorithms above are coded in MATLAB and run on a dual-core PC with 3.10 GHz Intel Core i5-2400 and 4 GB RAM.

### 4.1. Comparison of Different Regularization Parameters

In the following experiments, all the parameters except the regularization parameter *λ* keep invariant for the same phantom with the same projection noise level. Three values of *λ* are selected, of which one is calculated by the proposed algorithm, another one is multiplied by 0.1, and the third one is multiplied by 10. The distance from the X-ray source to the center point of the phantom is twice the length of the image edge. The iteration of the algorithm is stopped when the relative error err^*δ*^ = |*δ* ^*t*^ − *δ* ^*t*−1^|/*δ* ^*t*^ is less than a stopping value (*δ* is calculated by (24)).

**Figure 2:**
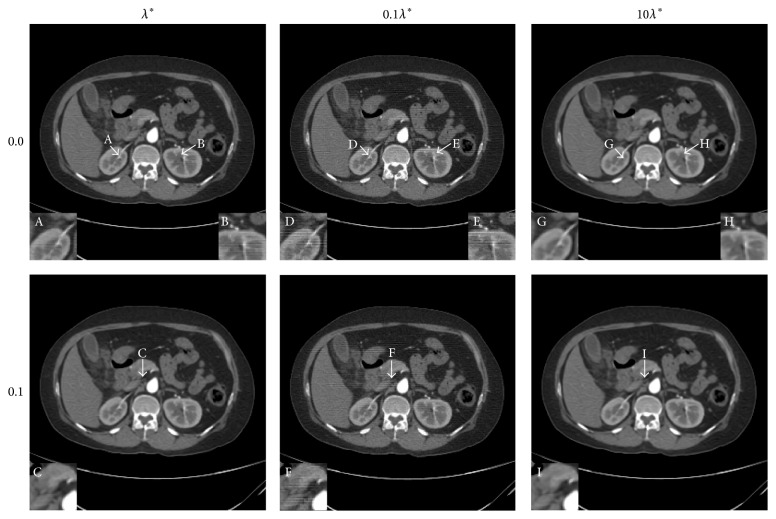
The results of human abdomen slice simulation study. From top to bottom, the noise levels are 0.0% and 0.1% in turn. From left to right, the regularization parameters are *λ* ^*∗*^, 0.1*λ* ^*∗*^, and 10*λ* ^*∗*^. The display window is [−160,400] HU.

**Figure 3:**
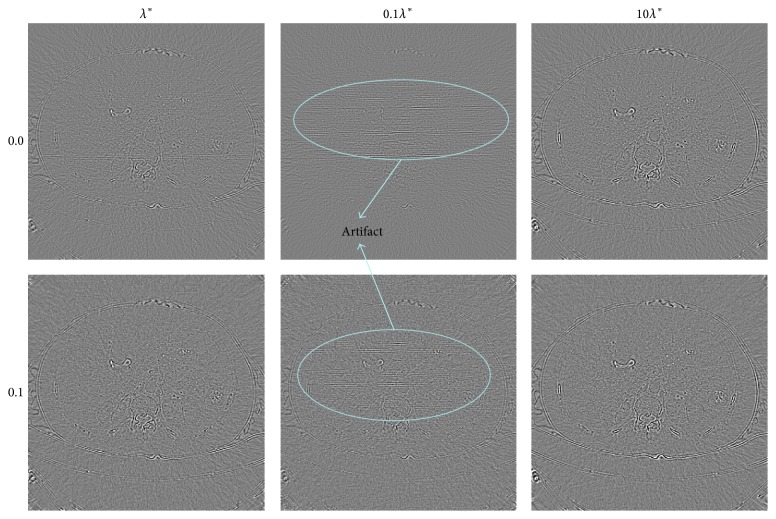
The difference between the reconstructed image and the original image (OI) of the human abdomen slice image. From top to bottom, the noise levels are 0.0% and 0.1% in turn. From left to right, the regularization parameters are *λ* ^*∗*^, 0.1*λ* ^*∗*^, and 10*λ* ^*∗*^. The display window is [−90,90] HU.

To compare the difference between different selections of the regularization parameter, the human abdomen slice image is tested as an example. The projection data are simulated by 180 views of 2° step length over a 360° range, and 512 detector elements are distributed in fan-beam geometry covering the phantom. The noise levels added to the projection data are 0.0% and 0.1% Gaussian random noise. The results are displayed in Figures 2 and 3. For the reason that biomedical images are often observed by a proper window to find more details, the images are displayed with a window [−160,400] HU. The difference between the reconstructed image and the phantom image is displayed by a window [−90,90] HU.

There are two criterions to evaluate the reconstructed image. One is the normalized mean absolute deviation (NMAD), defined as

(28) $$\begin{array}{c} NMAD\left(\;\%\;\right)=\frac{\sum_{i,j}^{}\left|\mu_{ij}-\mu_{ij}^{\text{truth}}\right|}{\sum_{i,j}^{}\left|\mu_{ij}^{truth}\right|}\times 100. \end{array}$$

The other one is the signal-to-noise ratio (SNR), defined as

(29) $$\begin{array}{c} \text{SNR}=10\mathrm{lg}\left(\;\frac{\sum_{i,j}^{}{\left(\mu_{ij}^{\text{truth}}\right)}^{2}}{\sum_{i,j}^{}{\left(\mu_{ij}-\mu_{ij}^{\text{truth}}\right)}^{2}}\;\right). \end{array}$$

The values of the two criterions are presented in Table 1. Comparing the results with the same noise level of the *λ* situation and the 10*λ* situation, the NMAD of the *λ* situation is smaller and the SNR of the *λ* situation is larger mostly, which proves that the image reconstructed by choosing the regularization parameter as *λ* is more close to the sample image. When it comes to the 0.1*λ* situation, although the values of the two criterions are a little better, there are some artifacts appearing in the reconstructed image, leading to a decline of the image quality. In the middle column of Figures 2 and 3, some horizontal line artifacts appear in the images (regions D, E, and F in Figure 2 and the ellipse regions in Figure 3). It seems that there are more horizontal artifacts in the 0.0% noise level image. The probable reason is the noise added to the projection data since the noise covers the inconspicuous artifact in the 0.1% noise level image. So what is the reason for the fact that the NMAD and SNR of the 0.1*λ* situation are better? One reasonable explanation might be the smoothing effect of the dictionary learning algorithm. When the iterative image is updated by (6) or (23), the sparse constraint added by dictionary learning method smoothes not only the noise but also the margin details. This effect becomes more significant when the regularization parameter is becoming larger. Therefore, the difference between the reconstructed image and the sample image in the margin area becomes more obvious, which is displayed in the left and right columns of Figure 3. By discovering this effect making the criterion worse, future work should be devoted to improving the reconstruction algorithm based on dictionary learning in order to smooth the noise and preserve the margin details meanwhile.

### 4.2. Comparison of Different Reconstruction Algorithms

**Figure 4:**
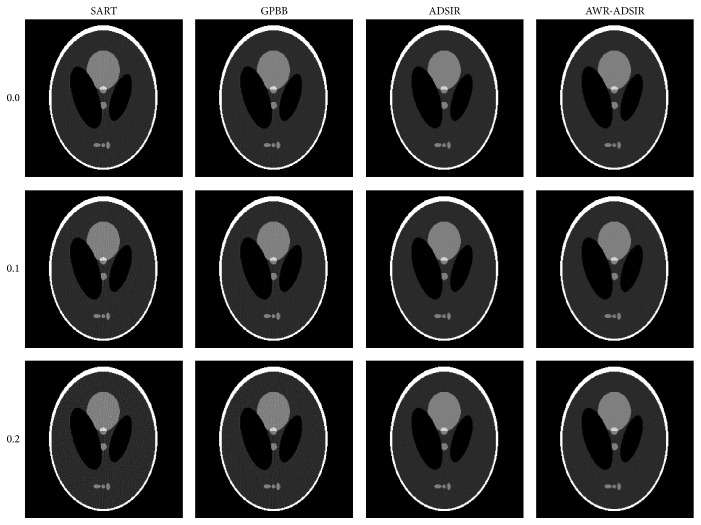
Reconstructed images from low-dose projection data of the Shepp-Logan phantom. From top to bottom, the noise levels are 0.0%, 0.1%, and 0.2% in turn. From left to right, the reconstruction algorithms are SART, GPBB, ADSIR, and AWR-ADSIR, respectively. The display window is [0.15,0.45].

To present the advantage of AWR-ADSIR, the images reconstructed by AWR-ADSIR and some other algorithms (SART, GPBB, and ADSIR) are compared with the same projection data, initial conditions, and stopping criterions. The testing examples are the Shepp-Logan phantom and the human head slice image. The Shepp-Logan phantom is simulated by 120 views of 3° step length over a 360° range, and 512 detector elements are distributed in fan-beam geometry with three different noise levels while the head slice sample is simulated by 180 views of 2° step length over a 360° range. The reconstructed results are displayed in the four figures (Figure 4 to Figure 7).

**Figure 5:**
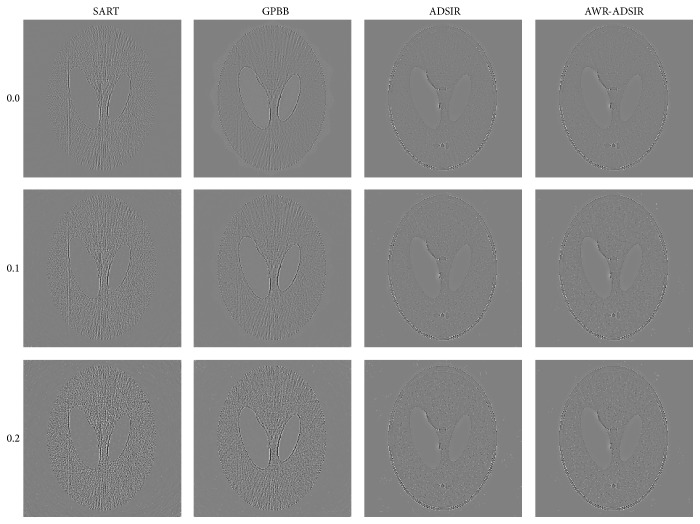
The difference between the reconstructed image and the original image (OI) of the Shepp-Logan phantom. From top to bottom, the noise levels are 0.0%, 0.1%, and 0.2% in turn. From left to right, the reconstruction algorithms are SART, GPBB, ADSIR, and AWR-ADSIR, respectively. The display window is [−0.05,0.05].

**Figure 6 fig6:**
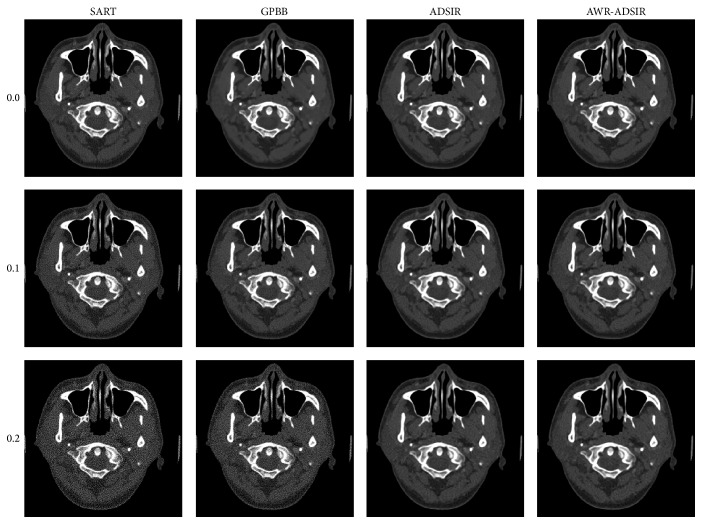
The results of human head slice simulation study. From top to bottom, the noise levels are 0.0%, 0.1%, and 0.2% in turn. From left to right, the reconstruction algorithms are SART, GPBB, ADSIR, and AWR-ADSIR, respectively. The display window is [400,1000] HU.

**Figure 7:**
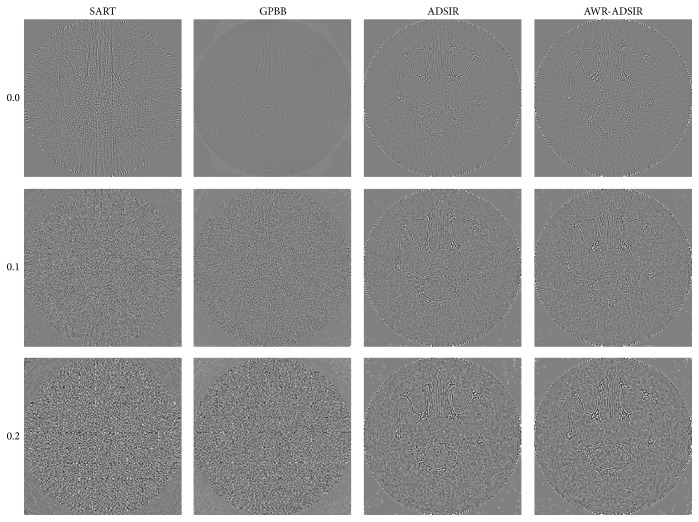
The difference between the reconstructed image and the original image (OI) of the human head slice image. From top to bottom, the noise levels are 0.0%, 0.1%, and 0.2% in turn. From left to right, the reconstruction algorithms are SART, GPBB, ADSIR, and AWR-ADSIR, respectively. The display window is [−100, 100] HU.

With the comparative results calculated by (28) and (29) presented in Tables 2 and 3, the quality of all the reconstructed images is decreased with the noise level increasing. Among these four algorithms, SART generates the worst results. GPBB behaves well when the noise level is very low but when the noise level is beyond 0.1%, the quality of reconstructed image degenerates quickly. The regularization parameter in ADSIR is empirically selected according to [12]. The fact that the image quality and comparing criterions of ADSIR and AWR-ADSIR are mostly the same proves that the parameter selecting model in AWR-ADSIR is practical and efficient. In addition, the marginal details appearing in Figures 5 [Fig fig6] and 7 of algorithms ADSIR and AWR-ADSIR indicate the smoothing effect on the margins again. Since the high-contrast edge is smoothed by the algorithm, the structural boundaries appear in the difference image in Figure 7. Obviously, when the empirical regularization parameter is unknown, the ADSIR algorithm costs a large amount of time to determine the proper value by repeatedly operating the iterative process (usually more than ten times) while the AWR-ADSIR algorithm only operates the iterative process twice as Algorithm 1 shows. The model indeed reduces the time consumption to determine the value of the regularization parameter.

### 4.3. Adaptive Weight Regularized GDSIR

**Figure 8:**
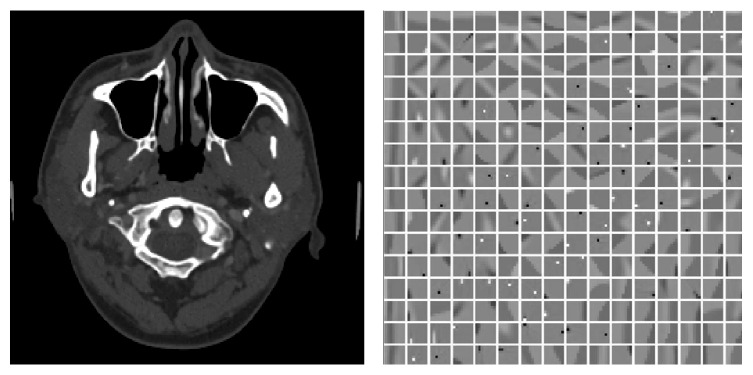
The image reconstructed by AWR-GDSIR and the global dictionary. The dictionary is displayed in window [−1,1], which is constructed training based on the patches extracted from original image. The image is reconstructed by 0.0% noise level projection data displayed in window [400,1000] HU.

The last experiment is applying the parameter selecting strategy into the GDSIR model. The dictionary displayed in Figure 8 is learned from the overlapping patches of the original image in Figure 1. The reconstruction process of AWR-GDSIR is almost the same as AWR-ADSIR except that the dictionary has been constructed in advance and dose not change during the reconstruction process. The result shown in Figure 8 indicates that the proposed model is also suitable for the general dictionary condition.

## 5. Conclusion

In most optimization problems, the determination of the regularization parameter is still a problem. In this paper, aiming to determine the regularization parameter of the algorithm based on dictionary learning, one model function, whose independent variable *δ*_*λ*→*∞*_ can be calculated by the known projection data, is proposed depending on some modification on the objective function. When compared to some other algorithms, the images reconstructed by AWR-ADSIR and ADSIR are of similar quality, better than the one reconstructed by SART, and the proposed algorithm is much more robust to noise than GPBB. This indicates that the modification of the objective function does not degrade the performance of ADSIR. What is more, the parameter selection model is demonstrated to be rational by the fact that the image quality of the *λ* situation is better than the ones of 0.1*λ* and 10*λ* situations. However, when some other parameters (like the scale of the dictionary, the scale of the patch, and so on) change, the model function might result in some difference. However, it still works to look for the function relationship between these two parameters since the monotonous relation between *δ*_*λ*→*∞*_ and *λ* remains.

By validating the proposed selecting principle, the smoothing effect on image margins is discovered. Our future work will focus on improving the dictionary learning method, with the expectation to maintain the smoothing effect on noise regions and preserve marginal information better.

## Figures and Tables

**Algorithm 1:**
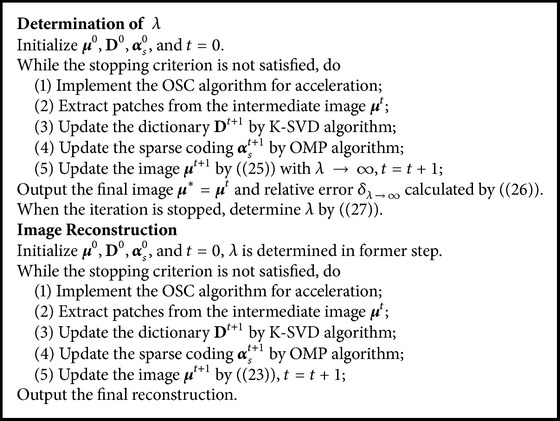
Workflow of the developed algorithm.

**Table 1:** Quantitative evaluation of the results with different regularization parameters.

Noise level	*δ*_*G*_ (10^6^ *δ*_*λ*→∞_)	*λ*		NMAD (%)	SNR (dB)
0.0%	1.7094	0.9005	*λ*	1.5819	35.5246
			0.1*λ*	1.2406	37.6730
			10*λ*	2.0147	32.9517

0.1%	2.5269	5.7462	*λ*	2.0371	33.1220
			0.1*λ*	1.8742	34.5110
			10*λ*	2.1561	32.4527

**Table 2:** Comparing criterions of the results reconstructed by different algorithms (Shepp-Logan).

Algorithm	Criterion	Noise level		
		0.0%	0.1%	0.2%
SART	NMAD (%)	2.1510	2.9273	4.1032
	SNR (dB)	33.5448	31.5220	28.7997
GPBB	NMAD (%)	1.3472	1.7826	3.0473
	SNR (dB)	36.5443	34.6140	30.5004
ADSIR	NMAD (%)	0.8110	0.9553	1.1823
	SNR (dB)	35.7740	34.8516	33.3413
AWR-ADSIR	NMAD (%)	0.8223	1.0639	1.1549
	SNR (dB)	35.5354	34.5480	33.7801

**Table 3:** Comparing criterions of the results reconstructed by different algorithms (head).

Algorithm	Criterion	Noise level		
		0.0%	0.1%	0.2%
SART	NMAD (%)	1.0904	2.2017	4.0009
	SNR (dB)	36.8554	31.6575	26.4913
GPBB	NMAD (%)	0.4837	1.1631	2.7798
	SNR (dB)	45.0405	36.7565	29.4104
ADSIR	NMAD (%)	0.5981	0.9370	1.1693
	SNR (dB)	39.0515	34.9548	32.8972
AWR-ADSIR	NMAD (%)	0.6765	0.9438	1.1572
	SNR (dB)	37.7225	34.9687	32.9703
